# Dynamic and Assembly of Epiphyte and Endophyte Lactic Acid Bacteria During the Life Cycle of *Origanum vulgare* L.

**DOI:** 10.3389/fmicb.2018.01372

**Published:** 2018-06-26

**Authors:** Erica Pontonio, Raffaella Di Cagno, Waed Tarraf, Pasquale Filannino, Giuseppe De Mastro, Marco Gobbetti

**Affiliations:** ^1^Department of Soil, Plant and Food Sciences, University of Bari Aldo Moro, Bari, Italy; ^2^Faculty of Science and Technology, Libera Università di Bolzano, Bolzano, Italy; ^3^Department of Agricultural and Environmental Sciences, University of Bari Aldo Moro, Bari, Italy

**Keywords:** epiphyte bacteria, endophyte bacteria, oregano, lactic acid bacteria, essential oils, thymol, carvacrol

## Abstract

*Origanum vulgare* L. (oregano) was chosen as suitable model to investigate the ability of the endophyte-microbiome, especially that of lactic acid bacteria, to develop specific interactions with the plant, mediated by the essential oils (EOs). Combined culture-dependent and -independent approaches analyzed the bacterial dynamic and assembly of *Origanum vulgare* L. throughout the life cycle. Epiphyte bacteria were more abundant than the endophyte ones. The number of presumptive lactic acid bacteria increased throughout oregano life cycle, according to the plant organ. Diverse species of lactic acid bacteria populated the plant, but *Lactobacillus plantarum* stably dominated both epiphyte and endophyte populations. High-throughput DNA sequencing showed highest epiphyte bacterial diversity at early vegetative and full-flowering stages, with blooming signing the main microbial differentiation among plant organs. *Proteobacteria*, *Actinobacteria* and *Bacteroidetes*, and *Firmicutes* and *Cyanobacteria* at lower abundance were the main phyla. Various genera were detectable, but oregano harbored mainly *Methylobacterium*, *Sphingomonas*, *Rhizobium* and *Aurantimonas* throughout phenological stages. *Firmicutes* epiphyte and endophyte microbiotas were different, with a core microbiota consisting of *Bacillus*, *Exiguobacterium*, *Streptococcus*, *Staphylococcus* and *Lactobacillus* genera. *Bacillus* dominated throughout phenological stages. High-throughput DNA sequencing confirmed the dominance of *L. plantarum* within the epiphyte and endophyte populations of lactic acid bacteria. Yields of EOs varied among plant organs and throughout plant life cycle. *L. plantarum* strains were the most resistant to the total EOs (mainly thymol and carvacrol) as extracted from the plant. The positive correlation among endophyte lactic acid bacteria and the EOs content seems confirm the hypothesis that the colonization within plant niches may be regulated by mechanisms linked to the synthesis of the secondary metabolites.

## Introduction

Plant-associated microenvironments interact with a diversity of beneficial and pathogenic microbes, which are of pivotal importance for plant nutrition, healthy status and defense. Roughly, it has estimated that the one billion square kilometers of worldwide leaf surfaces hosts more than 10^26^ bacteria (Vorholt, 2012). Although this immeasurable arsenal of microbes, each plant harbors a specific microbiota. The composition and size of this depends on numerous biotic and abiotic drivers: plant species, phenological stage, climate and season, spatial distribution and nutrient availability (Berg and Smalla, 2009; Barnard et al., 2013).

Within plant diversity, medicinal and aromatic species harbor distinctive microbiomes because of their unique and structurally divergent secondary metabolites (Qi et al., 2012; Schmidt et al., 2014). Interactions between medicinal and aromatic plants and microbes have fascinated scientists all over the world since the plant-associated microbiome, especially the endophyte-microbiome, is presumably responsible, directly or indirectly, for the synthesis of bioactive phytochemicals (Köberl et al., 2013).

*Origanum vulgare* L. (oregano) is morphologically the most variable species within the genus *Origanum*, which belongs to the *Lamiaceae* family. Oregano is widely distributed throughout Mediterranean and Asia areas (Kintzios, 2002). Even popular as a culinary herb, anciently oregano was a medical plant in many countries (Souza et al., 2007). Recently, it deserves a marked interest for cosmetic and perfumery industries due to its spicy fragrance (Pande et al., 2012). The high levels of volatile constituents (essential oils, EOs) (Esen et al., 2007; Karakaya et al., 2011), flavonoids and phenolic acids (Liang et al., 2012) are responsible for the medical activities and, more in general, for the multifaceted industrial interest. Thymol and carvacrol are the major constituents of oregano EOs, with a remarkable inhibitory activity against microbes (Lambert et al., 2001; Bassolé and Juliani, 2012). Plant genotype and environmental conditions both affect the synthesis of these natural metabolites (Sharafzadeh and Ordookhani, 2011). Living in association with plant cells that synthesize EOs, endophyte bacteria have to tolerate these chemical compounds and further may act as biotic drivers with an active role in the biotransformation (Monteiro et al., 2009; Tiwari et al., 2010). Endophytes, inhabiting inter- or intra-cellular host tissues, synthesize secondary metabolites, which enhance the ecological plant fitness.

Overall, plant microbiology has mainly focused on common symbiotic rhizosphere microbes (Vessey, 2003) and pathogens (Bais et al., 2006). To date, reports on the microbiota of oregano have concerned only the root microenvironment (Bafana, 2013). The functional role of specific microbial groups, including lactic acid bacteria that inhabit the oregano plant, still needs a deeper investigation. Lactic acid bacteria, and especially lactobacilli, are present in the phyllosphere, endosphere and rhizosphere of many plants (Lamont et al., 2017). The ability of lactic acid bacteria to live in the plant endosphere suggests an intimate relationship, which is responsible to enhance plant production by improving nutrient availability, acting as a biocontrol agent, alleviating biotic and biotic stresses, and directly stimulating plant growth (Lamont et al., 2017). Recently, the definition of plant growth promoting microbes, which paired with the GRAS status (Lamont et al., 2017), has attributed to lactic acid bacteria. A key question still open related to interface plant-bacteria is also the adaptation of the endophytes to plant secondary metabolites, especially to those exerting antibacterial activity. For instance, plants accumulating toxic molecules such as heavy metals harbor bacteria resistant to these toxic compounds (Mengoni et al., 2010). Medicinal and aromatic plant species producing EOs were considered as a suitable model for testing the hypothesis of an effect of the endophyte-microbiome on the production of EOs and, consequently of a colonization of plant tissues by bacteria resistant to these oils (Checcucci et al., 2017).

The level of the EOs and the dynamic of epiphyte and endophyte bacteria from the early stages of colonization of the young oregano plant to the assembled communities in mature plant, including their spatial distribution, is unknown. To show and to follow the presence of oregano-associated lactic acid bacteria simultaneously with the progress of the EOs is the first and indispensable step for exploiting their potential role during the plant cycle life.

This study describes the microbiome dynamic and assembly during the entire life cycle of oregano plant and how these may be linked to the synthesis of the EOs. For this purpose, chemical analysis targeting the EOs and a combination of culture-dependent and -independent approaches analyzed the bacterial community structure and diversity of oregano plant during the entire life cycle. The assessment of the lactic acid bacteria resistance toward EOs completed the study.

## Materials and Methods

### Plant Material

A genotype of *Origanum vulgare* L., which was selected clonally based on agronomic features and EO yield and composition (De Mastro et al., 2004), was cultivated (fall 2014) in the experimental farm “Enrico Pantanelli” of the University of Bari “A. Moro” located in Policoro (Basilicata, Italy; 40°10′20″ N, 16°39′04″ E) at an altitude of 15 m above sea level. This site is 15 m above sea level and is characterized by a Mediterranean climate according to the De Martonne classification (Cantore et al., 1987) with an average annual rainfall of ca. 560 mm distributed mainly during autumn and winter. The soil, more than 1.2 m deep, has a standard loam texture according to the physical characteristics: sand 398 g/kg, silt 374 g/kg clay 228 g/kg. Soil chemical characteristics were: pH 7.7; total N (Kjeldahl method) 1.7 g/kg/, available P_2_O_5_ (Olsen method) 27.6 mg/kg, exchangeable K_2_O (ammonium acetate method) 227 mg/kg, organic matter 2.3 % (G.U. Suppl. Ordin. n° 248, 21/10/1999), total carbonates 110.2 g/kg, active carbonate 55.7 g/kg; saturated paste extract electrical conductivity (ECe) 0.95 dS/m, ESP 1.9%; bulk density 1.25 kg dm^−3^; soil moisture at field capacity (measured *in situ*) 31.5% and at wilting point (−1.5 MPa) 15% of soil dry weight. Meteorological conditions (rainfall and mean air temperature) were monitored during the trial by a meteorological station sited at the experimental farm. Harvesting of plants was at the following phenological stages: early vegetative (acronym used, I), late vegetative (II), blooming (III), and full-flowering (IV) (Supplementary Figure S1). For each sampling time, a group of 10 plants was cut uniformly at 10 cm above ground to ensure regrowth. During phenological stages, epigeous plant organs (phyllosphere) from the group were separated into leaves, stems and flowers by using a sterile scalpel (Supplementary Figure S1). Pooled plant organs were inserted into Whirl-Pak sterile sampling bags (Nasco, Fort Atkinson, WI, United States), kept at cool temperature, and laboratory processed within 4 h from collection. Three replicates of pooled plant organs were used for all the analyses, which were performed in triplicates.

### Enumeration of Cultivable Epiphyte and Endophyte Bacteria

Ten-gram of pooled and washed plant organs (leaves, stems or flowers) were immersed into 200 ml of DNA-free 0.1 M potassium phosphate buffer (pH 7.0) in a 500-ml Erlenmeyer flask and treated (7 min at 30°C) in an ultrasonic bath (RK103H Sonorex Super, Ultra Center Europe, Zwolle, Netherlands) (Minervini et al., 2015). Plant organs were taken out of the flask for further treatment, whereas epiphytic microorganisms released into the buffer were pelleted by centrifugation (20,000 × *g* for 30 min at 4°C) and then re-suspended in 5 ml of potassium phosphate buffer (Minervini et al., 2015). Afterward, plant organs were surface sterilized by soaking in 150 ml of a 15% [vol/vol] H_2_O_2_ solution, and then shacked for 15 min by a gentle rotary, protecting from light. After shacking, plant organs were washed twice in sterile demineralized water and, finally dried in a laminar flow hood for 1 h (Minervini et al., 2015). Surfaces of plant organs were assessed for sterility by blotting them tightly on 1/10-strength tryptic soy agar and incubating plates at 30°C for 48 h (Minervini et al., 2015). No microbial growth was found in 10 g of sample. Surface-sterilized plant organs were homogenized (3 min) with 80 ml of 0.1 M potassium phosphate buffer (pH 7.0) in a BagMixer 400P (Interscience, Saint Nom, France) blender. The homogenate, free of plant debris, contained endophyte microorganisms. One milliliter of epiphytic or endophytic suspension was used to enumerate total aerobic bacteria and presumptive lactic acid bacteria. Total aerobic bacteria were enumerated on BYS (0.1% [wt/vol] of bactopeptone, 0.1% [wt/vol] of yeast extract, 0.5% [wt/vol] of saccarose and 1.5% [wt/vol] of agar), supplemented with 0.1 g/l of cycloheximide (Sigma Chemical Co., United States) under aerobic conditions at 30°C for 48 h. Presumptive lactic acid bacteria were counted on MRS agar (Oxoid Ltd, Basingstoke, Hampshire, United Kingdom), supplemented with 0.1 g/l of cycloheximide (Sigma Chemical Co., United States). Plates were incubated under anaerobiosis (AnaeroGen and AnaeroJar; Oxoid, Basingstoke, Hampshire, United Kingdom) at 30°C for 48 h.

### Isolation of Epiphytic and Endophytic Lactic Acid Bacteria

Due to the low cell density of lactic bacteria in oregano, and in order to increase the chance to isolate different species and strains, enriched MRS broth containing 0.1% [wt/vol] of cycloheximide was also used for isolation (Chen et al., 2005). Ten milliliters of epiphytic or endophytic suspensions of each plant organ were suspended in 90 mL of MRS broth (Oxoid Ltd, Basingstoke, Hampshire, United Kingdom), and incubated at 30°C for 48 – 72 h under stirring conditions (100 rpm). Serial dilutions were made and plated on MRS agar (Oxoid) at 30°C for 48 h. At least 15 colonies of presumptive lactic acid bacteria were randomly selected from the plates containing the two highest sample dilutions. Gram-positive, catalase-negative, non-motile rod and coccus isolates were cultivated in MRS broth at 30°C for 24 h, and re-streaked onto the same agar medium. All isolates considered for further analysis were able to acidify the culture medium.

### Genotypic Characterization by Randomly Amplified Polymorphic DNA-Polymerase Chain Reaction (RAPD-PCR) Analysis

Genomic DNA from lactic acid bacteria was extracted using a DNeasy blood and tissue kit (Qiagen, SA, Courtaboeuf, France), according to the manufacturer’s instructions (Ahmed et al., 2009). Three oligonucleotides, P4 (5′-CCGCAGCGTT-3′), P7 (5′-AGCAGCGTGG-3′) (Corsetti et al., 2003) and M13 (5′-GAGGGTGGCGGTTCT-3′) (Stendid et al., 1994), with arbitrarily chosen sequences, were used for biotyping of lactic acid bacteria isolates. Reaction mixture and PCR conditions for primers P4 and P7 were those described by Corsetti et al. (2003), whereas those reported by Zapparoli et al. (1998) were used for primer M13. RAPD-PCR profiles were acquired by the MCE-202 MultiNA microchip electrophoresis system (Shimadzu s.r.l., Milan, Italy), using the DNA-2500 reagent kit (100–2500 bp) and the 2-log DNA ladder (0.1–10.0 kb) (Promega Srl, Padova, Italy) according to the manufacturer’s instructions. RAPD-PCR was also applied to identify unique populations. The similarity of the electrophoretic profiles was assessed by determining the Dice coefficients of similarity and using the unweighted-pair group method using average linkages (UPGMA) algorithm.

### Genotypic Identification of Lactic Acid Bacteria

To identify presumptive lactic acid bacterial strains two primer pairs (Sigma Chemical Co. Milan, Italy), LacbF/LacbR and LpCoF/LpCoR (Sigma Chemical Co. Milan, Italy), were used for amplifying the 16S rRNA genes (De Angelis et al., 2006). The expected amplicons of ca. 1400 and 1000 bp were eluted from the gel and purified by the Nucleospin gel and PCR clean-up kit (Macherey–Nagel, Düren, Germany). PCR products were separated by electrophoresis, purified as described above, and subjected to Sanger sequencing (Sanger et al., 1977). Taxonomic strain identification was performed by comparing the sequences of each isolate with those reported in the NCBI Reference Sequence (RefSeq) database (Altschul et al., 1997). Identifications were confirmed using EZ-TAXON database^1^ (Park et al., 2012). Strains showing homology of at least 97% were considered to belong to the same species (Goebel and Stackebrandt, 1994). Cultures were maintained as stocks in 15% (v/v) glycerol at −80°C and routinely propagated at 30°C for 24 h in MRS broth.

### DNA Extraction From Plant Organs

Suspensions of epiphytic and endophytic microbes were prepared as reported above and used as starting material for total DNA extraction. Total genomic DNA was extracted from 0.5 ml of suspensions containing either epiphytic or endophytic microorganisms, using the FastDNA Pro Soil-Direct kit (MP Biomedicals, Santa Ana, CA, United States) coupled to the FastPrep instrument (MP Biomedicals), according to the manufacturer’s instructions. Quality and concentration of DNA extracts were assayed by spectrophotometric measurements using a NanoDrop ND1000 (Thermo Fisher Scientific Inc., Marietta, OH, United States). DNA extraction was carried out in triplicate on three replicates of each pool of plant organs.

### Illumina MiSeq Analysis

DNA extracted from three different replicates were pooled (Middelbos et al., 2010; Minervini et al., 2015) and used as template for Illumina MiSeq 2x300 diversity analyses, which were carried out at the Research and Testing Laboratory (RTL; Lubbock, TX, United States). Pooled DNA samples were prepared, such that each individual sample contributed an equal amount of DNA (Middelbos et al., 2010). Primers targeting the V1–V3 region (Kim et al., 2011; Zheng et al., 2015; Allen et al., 2016) (*Escherichia coli* position 28–519, forward 28F: GAGTTTGATCNTGGCTCAG and reverse 519R: GTNTTACNGCGGCKGCTG) of the 16S rRNA gene (Reeder and Knight, 2010) were used for bacterial assay. Primers Firm350F and Firm814R were used to amplify a fragment of the 16S rRNA gene for analysis of diversity inside the phylum of Firmicutes (Minervini et al., 2015). PCR and pyrosequencing procedures were carried out based upon RTL protocols http://www.researchandtesting.com (Lubbock, TX, United States). Illumina MiSeq analysis.

### Bioinformatics

Sequenced reads for each sample were processed through denoising and chimera detection by using Research and Testing Laboratory’s in-house pipeline, described at https://static1.squarespace.com/static/5807c0ce579fb39e1dd6addd/t/5813af0fd482e97e5eb4fcb5/1477685010205/Data_Analysis_Methodology.pdf. Briefly, sequences were grouped using their barcodes and any sequence that contained a low quality barcode or that failed to be at least half the expected amplicon length (or 250 bp, whichever was shortest) was removed from the data pool. Sequences that passed the quality filter were denoised using an algorithm based on USEARCH pipeline (Edgar, 2010) (prefix dereplication) into clusters (4% dissimilarity among sequences of the same cluster), so that each sequence of shorter length to the centroid sequence must be a 100% match to the centroid sequence for the length of the sequence. Following denoising sequences were checked for chimeras using (Edgar, 2010). Finally, sequence data were separated into operational taxonomic units (OTUs) at 97% similarity using a USEARCH and all OTUs were used for classification by using UBLAST global alignment against a custom16S database comprised of well characterized sequences from nr/nt. Each sequence was corrected base by base in order to remove noise. The output was then analyzed using a internally developed Python pipeline that parses the assigned taxonomic information to create the final analysis files. Alpha- and beta-diversities were evaluated by QIIME, as recently described (De Filippis et al., 2013).

### Essential Oils (EOs) Analysis

The extraction and analysis of EOs were carried out in triplicate on three replicates of each pool of plant organs. Aerial parts of plants collected during the various phenological stages were dried through a forced convection oven (38°C) until constant weight was achieved, and then were subjected to hydro-distillation for 4 h using a Clevenger apparatus. The collected EOs were dried over anhydrous sodium sulfate and stored in a dark glass vial, at 4 °C until further analysis. The EOs percentage was expressed as v/w based on dry matter of the initial material. Analysis of EOs was performed using a Gas Chromatography system (GC) Hewlett Packard 6890 equipped with a HP-5 MS capillary column (5% – phenylmethypolysiloxane, 0.25 μm × 30 m × 0.25 μm film thicknesses) and a mass selective detector (MSD) HP 5972. For GC–MS detection, an electron ionization system with ionization energy of 70 eV was used. Helium was the carrier gas, at a flow rate of 1.1 ml/min. Injector and MS transfer line temperatures were set at 250 and 300°C, respectively. Column temperature was initially kept at 60°C for 3 min, then gradually increased to 110°C at a 2°C/min rate, held for 10 min and finally raised to 220°C at 10°C/min. EOs were identified based on the comparison of their relative retention time and mass spectra with those of the library data of the GC–MS system (Adams, 2001) and literature data (Wiley Registry of Mass Spectral Data, 1995). Results were also confirmed by injection of a mixture of aliphatic hydrocarbons (C8 - C32) (Sigma, Milan, Italy) under the above conditions to calculate the retention index (RI) for each component.

### Minimum Inhibitory Concentration (MIC)

Minimum inhibitory concentration values were determined for the identified lactic acid bacterial strains. EO and whole plant extracts dissolved in 10% dimethyl sulfoxide [vol/vol], were filtered through 0.22 μm Millipore filters, and diluted in MRS broth to the highest concentration (1000 μg/ml) to be tested. Then, serial twofold dilutions were made in order to obtain a concentration range from 1000 to 3.9 μg/ml in 10 ml sterile test tubes containing MRS broth. Microorganisms were inoculated into each tube at the final density of ca. 5 Log cfu/ml. A control test containing broth supplemented with only EO was also performed. Tubes were then incubated at 30°C for 24 h. Prior and after incubation, bacterial cell density was determined by measuring the optical density at 620 nm (OD_620_). The data were collected from three independent experiments The MIC was defined as the lowest concentration of the EO inhibiting the bacterial growth after incubation for 24 h.

### Statistics

Three replicates of pooled plant organs were used for enumeration of cultivable bacteria, EOs extraction and analysis, and total DNA extraction. Each analysis was performed in triplicate. Data were subjected to one-way ANOVA, and pair-comparison of treatment means was achieved by Tukey’s procedure at *P* < 0.05, using a statistical software (Statistica 7.0 per Windows).

Weighted and unweighted UniFrac distance matrices and OTU tables were used to perform ADONIS and ANOSIM statistical tests through the compare_category.py script of qiime to verify the microbial populations in the different samples. EOs data that mainly (*P* < 0.05) differentiated plant organs during the plant life cycle were used as variables for principal-component analysis (PCA).

Correlations among descriptors or objects were calculated with the Correlation matrices function (Pearson correlation) of Statistica 7.0 and the permutation analysis (Euclidean distance) of PermutMatrix.

### Nucleotide Sequence Accession Number

16S rRNA gene sequences are available in the Sequence Read Archive of NCBI (accession numbers PRJNA347573).

## Results

### Bacterial Enumeration

**FIGURE 1:**
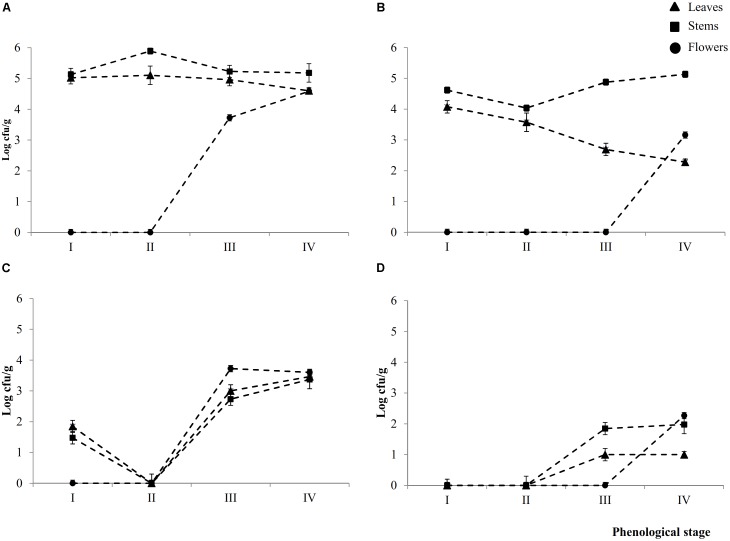
Cell numbers (Log cfu/ml) of total aerobic bacteria and presumptive lactic acid bacteria from epiphytic (**A,C**, respectively) and endophytic (**B,D**, respectively) fraction of leaves, stems and flowers during the early vegetative (I), late vegetative (II), blooming (III), and full flowering (IV) stages of *Origanum vulgare* L. plant. Data are the means of three replicates of pooled plant organs ± standard deviations (*n* = 3) analyzed in triplicate.

Epiphyte (Ep) and endophyte (En) total aerobic bacteria were detectable in leaves (L), stems (S), and flowers (F) throughout the phenological stages (I – IV) (**Figures 1A,B**). Cell densities of Ep inhabiting L and S attained the highest (*P* < 0.05) levels during early- (I) (5.0 ± 0.2 and 5.1 ± 0.1 Log cfu/ml, respectively) and late-vegetative (II) (5.1 ± 0.2 and 5.8 ± 0.1 Log cfu/ml, respectively) stages of the plant life cycle. During blooming (III) and full-flowering (IV) stages, the cell number in leaves slightly (*P* < 0.05) decreased, whereas it remained almost constant in stems.

The number of Ep total aerobic bacteria inhabiting flowers increased by ca. one log cycle from stages III (3.7 ± 0.1 Log cfu/ml) to IV. Compared to Ep, leaves contained a significant (*P* < 0.05) lower number of En total areobic bacteria, which already decreased (ca. one log cycle) from stage I. This decrease continued up to stage IV (ca. 2.3 log cycles). Stems showed an almost similar trend during the I and II stages, then the cell number increased. Flowers harbored En total aerobic bacteria only at stage IV (3.1 ± 0.1 log cfu/ml).

Except for stage II, where both presumptive Ep and En lactic acid bacteria were not detectable in 10 ml of sample, all plants organs harbored lactic acid bacteria during stages III and IV. Cell densities varied depending on spatial distribution (**Figures 1C,D**). Ep lactic acid bacteria slightly (*P* < 0.05) differed among leaves, stems and flowers. The numbers varied from 2.7 ± 0.1 Log cfu/ml (EpSIII) to 3.7 ± 0.2 Log cfu/ml (EpFIII). At stage I, Ep lactic acid bacteria were 1.8 ± 0.1 and 1.5 ± 0.1 Log cfu/ml in leaves and stems, respectively. Apart from the plant organ and phenological stage, the cell number of En lactic acid bacteria was lower (*P* < 0.05) than that of Ep (ca. 1.5 log cycles). Stems and, especially, flowers showed the highest numbers. The overall trend showed that cell numbers of presumptive Ep and En lactic acid bacteria increased throughout the life cycle of oregano plant. Lactic acid bacteria contribution as percentage of total bacterial counts varied according to Ep or En fractions. As regard to the Ep, the contribution increased through the plant life cycle. Indeed with the only exception of the early vegetative stage where lactic acid bacteria accounted for less than 40% in both leaves and stems, in the last two phenological stages they represent more than 50% of the total bacterial counts. In leaves and flowers lactic acid bacteria contributed the most (60–100%) to the total bacteria. In En fraction, lactic acid bacteria represented ca. 40% of the total counts regardless the phonological stage and organ plant. The only exception was represented by flowers, where all bacteria were lactic acid bacteria.

### Isolation and Identification of Lactic Acid Bacteria

A RAPD-PCR analysis was carried out on Gram-positive, catalase-negative, non-motile, cocci and rods able to acidify MRS broth (325 isolates) (**Table 1**). The reproducibility of the RAPD fingerprints was assessed by comparing the PCR products obtained from three separate cultures of the same strain. Primers M13, P7, and P4 generated different patterns, which were used for cluster analysis. As shown by clustering RAPD profiles, the diversity among the isolates ranged from ca. 2.5 to 80% (Supplementary Figure S2). Clusters gathered isolates with a maximum level of diversity of 20%. The number of clusters was 21. With a few exceptions, Ep and En separated based on the phenological stages and regardless the plant organ. *O. vulgare* L. harbored *Lactobacillus plantarum* (11 strains), *Enterococcus mundtii* (5), *Lactobacillus rossiae* (2), *Enterococcus faecium* (2), *Leuconostoc citreum* (1), *Lactococcus lactis* (1) and *Lactobacillus graminis* (1) (**Table 1** and Supplementary Figure S2). The RAPD-PCR profiles of epiphyte or endophyte strains belonging to the same species, but isolated from various plant organs, differed. The highest diversity was at stages I and IV. Stage I harbored Ep and En *L. plantarum* (EpS, EpL and EnL), *Leuc. citreum* (EnL), *E. feacium* (EpL and EnS), and *E. mundtii* (EpS). At stage II, species were *L. rossiae* (EpL and EpS) and *L. plantarum* (EnS and EnL). Later (stage III), *L. plantarum* was always isolated, followed by *E. mundtii* (EpL and EnL). At stage IV, oregano plant contained *L. plantarum* (EnL, EnF and EnS), *E. mundtii* (EnS, EpL, EpS and EpF), *Lc. lactis* (EpL and EpS), *L. graminis* (EpF) and *E. feacium* (EnL). Accounting for more than 50% already at stage I, *L. plantarum* represented more than 80% of the total endophytes. Its dominance was stable throughout the entire plant life cycle. Other species such as *Lc. lactis*, *L. graminis* and *L. rossiae* were isolated as epiphytes only.

**Table 1:** Species of epiphytic (Ep) or endophytic (En) lactic acid bacteria isolated from leaves (L), stems (S), and flowers (F) of *Origanum vulgare* L. plant during the early vegetative (I), late vegetative (II), blooming (III), and full flowering (IV) stages.

Closest relative and identity (%)^a^/number of strains	Source of isolation^b^	Accession number NCBI RefSeq database (number of cluster)^c^	Accession number EZ-TAXON data base
*Lactobacillus plantarum* (99–100%)/11	EpLI; EpSI; EnLI; EnLII; EnSII; EpLIII; EpSIII; EpFIII; EnLIII; EnSIII; EnFIII; EnLIV; EnSIV; EnFIV	KT626386.1 (1); KJ187149.1 (4-17-19); KJ187148.1 (5); KJ187133.1 (6-15); JN851776.1 (7); AB598965.1 (8); KJ187143.1 (9); LC119064.1 (12)	ACGZ01000098
*Enterococcus mundtii* (99–100%)/5	EpSI (NC); EpLIII; EnLIII; EpLIV; EpSIV; EnSIV (NC); EpFIV	KR078353.1 (NC); KT723002.1 (21); KX156237.1 (14); KT765838.1 (11); KR078353.1 (NC)	JXKV01000056
*Lactobacillus rossiae* (100%)/ 2	EpLII; EpSII	KP742816.1 (18); KJ187180.1 (20)	AKZK01000036
*Enterococcus faecium* (100%)/ 2	EpLI; EnSIII; EnLIV	KT626392.1 (3); KT626401.1 (16)	AJKH01000109
*Leuconostoc citreum* (99-100%)/ 1	EnLI	KT968364.1 (2)	AF111948
*Lactococcus lactis* (99%)/ 1	EpLIV; EpSIV	JN863615.1 (10)	BALX01000047
*Lactobacillus graminis* (99%) / 1	EpFIV	LC097076.1 (13)	AYZB01000012

*^a^Species showing the highest identity (%) to the strain isolated from different plant organs. The percentage of identity was that shown by performing multiple sequence alignments in BLAST. Identification was carried out by 16S rRNA, recA, or pheS gene sequencing. ^b^Source of isolation are described in Section “Materials and Methods” and Supplementary Figure S4. ^c^Numbers of RAPD-PCR clusters. NC, not clustered.*

### High-Throughput DNA Sequencing

High relative abundance of Unknown hits in the endophyte fractions, may be due to the homology of the universal 16S primers targeting hypervariable regions V1–V3 (27F, 338R, 519R) to chloroplast 16S (Rastogi et al., 2010). Thereof, the results described hereafter refer only to epiphytes. A total of 73,207, 65,957, and 36,546 quality-trimmed sequences of 16S rRNA gene amplicons were obtained for leaves (average length 497.3 bp), S (average length 492.0 bp) and flowers (average length 529.6 bp), respectively. Supplementary Table S1 reports the number of OTU, the Chao1, Shannon and Simpson indices, and the richness estimator (ACE). Leaves, followed by stems and flowers, showed the highest microbial diversity. Apart from the plant organ, stages I and IV contained the highest biodiversity. From stage II to III, the diversity indices decreased and became the highest at stage IV. Bacteria were also analyzed using three phylogeny-based beta-diversity measures (Supplementary Figure S3). The principal coordinate analysis (PCoA) based on the weighted UniFrac distance matrix differentiated leaves and stems at stages I and II. Blooming signed the microbial differentiation among plant organs, which enlarged at stage IV especially for flowers.

### Microbiota Structure and Changes

**FIGURE 2:**
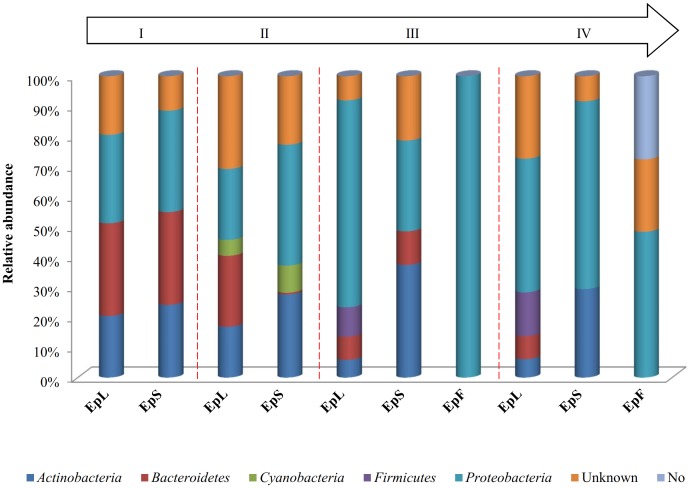
Relative abundance of bacterial phyla in DNA samples directly extracted from epiphytic (Ep) fraction of leaves (L), stems (S), and flowers (F) of *Origanum vulgare* L. plant during the early vegetative (I), late vegetative (II), blooming (III), and full flowering (IV) stages.

**FIGURE 3:**
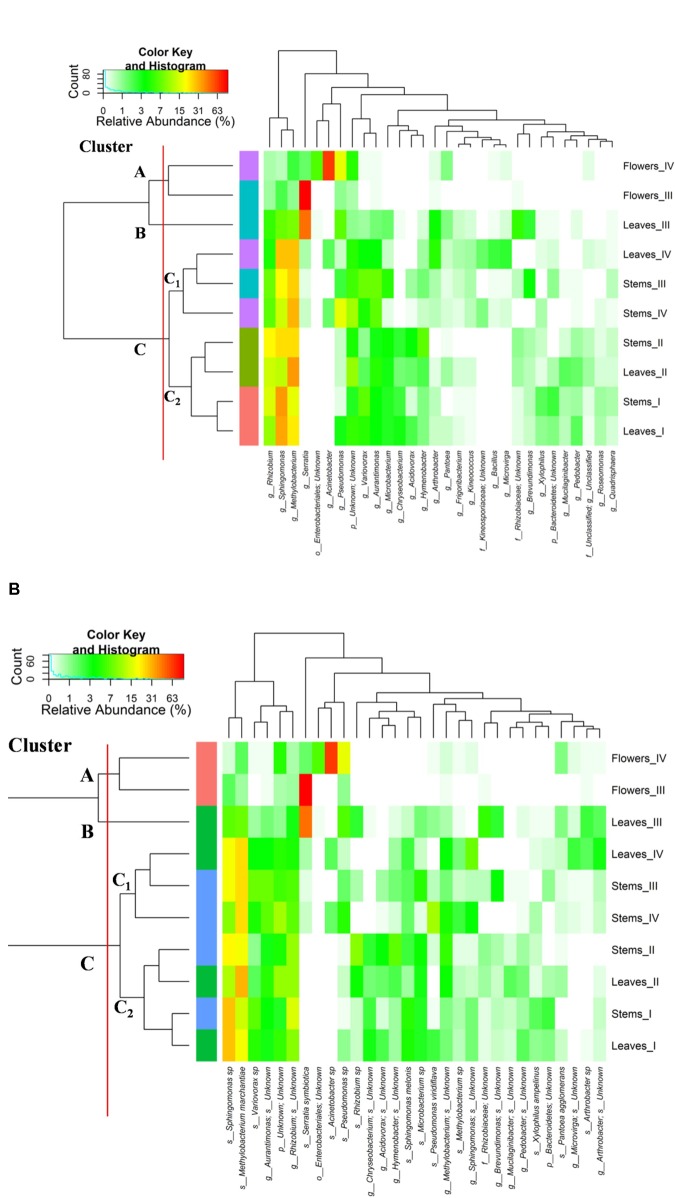
Pseudo heat map summarizing the relative abundances of the 30 most dominant genera **(A)** and species **(B)** in DNA samples directly extracted from epiphytic bacteria found on leaves, stems and flowers of *Origanum vulgare* L. plant during the early vegetative (I), late vegetative (II), blooming (III) and full-flowering (IV) stages. The color key defines the percentages of OTUs in the samples.

The relative abundances of bacterial sequences from DNA, which were assigned to bacterial phyla, varied. Spatial distribution, phenological stages and plant organs were the main drivers for diversity (**Figure 2**). Although with differences in the abundance, all plant organs harbored epiphyte *Proteobacteria*, *Actinobacteria*, and *Bacteroidetes*. At low abundance, leaves harbored *Firmicutes* in stages III and IV. At stage II, *Cyanobacteria* were present in leaves and stems. Leaves and stems harbored *Proteobacteria* and *Actinobacteria* throughout the entire life cycle. Only flowers harbored *Proteobacteria*. From stages I to IV, the relative abundance of *Bacteroidetes* decreased in leaves and stems. According to alpha- and beta-diversity, the epiphyte microbiota grouped into three main clusters (**Figure 3A**). Clustering was regardless the phenological stage. Cluster A grouped isolates from flowers at stages III and IV. Cluster B included the solely isolates from leaves at stage III. Cluster C split into sub-clusters C_1_ and C_2_, and grouped isolates from leaves and stems at stages IV, and I and II, respectively. Although with variable abundances, OTUs highlighted the persistence of some microbial genera (**Figure 3A**). Leaves, stems and flowers harbored *Methylobacterium* (8 – 34.0, 17 – 29, and 1 – 2%), *Sphingomonas* (7.6 – 32.5, 14 – 35, and 0.5 – 1.9%), *Rhizobium* (4.3 – 14.0, 6.8 – 19.6, and 0.6 – 0.8%) and *Aurantimonas* (1.3 – 4.8, 3.1 – 7.5, and 0.14 – 0.15%) throughout the phenological stages. *Microbacterium* and *Hymenobacter* were stably present (ca. 2 – 11.7%) in leaves and stems only. At stage III, *Serratia* dominated flowers but later disappeared. *Acidovorax* (5.5%) and *Chryseobacterium* (4.9%) occurred at stages I and II only. The majority of the genera present in leaves and stems belonged to α-*Proteobacteria* group. At stage IV, *Pseudomonas* (5.5%), *Acinetobacter* (20.7%), *Neokomagateae* (6.2%) and *Pantoea* (0.4%) from γ-*Proteobacteria* group, and the order Enterobacteriales (21.1%) were dominant in flowers. Although at very low abundances (up to 8.3%), genera from *Firmicutes* (e.g., *Bacillus*) populated leaves as epiphytes. *Methylobacterium marchantiae* was dominant in leaves and stems throughout the life cycle (**Figure 3B**). At stage III, flowers showed the presence of *Serratia symbiotica*. Other bacterial species (*Sphingomonas melonis*, *Pseudomonas viridiflava*, *Xylophilus ampelinus*, *Pantoea agglomerans*) were identified variously.

### *Firmicutes* Diversity

Total DNA from plant organs was amplified by *Firmicutes*-specific primers and subjected to high-throughput DNA sequencing. Epiphyte and endophyte microbiotas distinguished, obtaining 170,465 and 462,241 quality-trimmed sequences of 16S rRNA gene amplicons, respectively. The average number of sequences per sample was 17046.5 and 51360.11 with an average length of 487.83 and 471.6 bp, respectively. Good’s estimated sample coverage (median value of ca. 98%; *P* > 0.05) indicated the satisfactory coverage. Supplementary Table S2 reports the number of OTU, the Chao1 and Shannon, Simpson indices, and the richness estimator (ACE). Repeated amplifications of the DNA extracted from stems at stage III did not give reliable results, thus further excluding the sample coded as EnSIII. During oregano life cycle, stages I and, especially, IV had the highest microbial diversity. Regardless the plant organ, endophyte samples had the lowest biodiversity compared to the epiphyte counterpart. PCoA, based on the weighted UniFrac distance matrix, clearly differentiated endophyte samples. They featured with the lowest variability and aligned in the right part of the plot (Supplementary Figure S4).

### Core *Firmicutes* Microbiota

**FIGURE 4:**
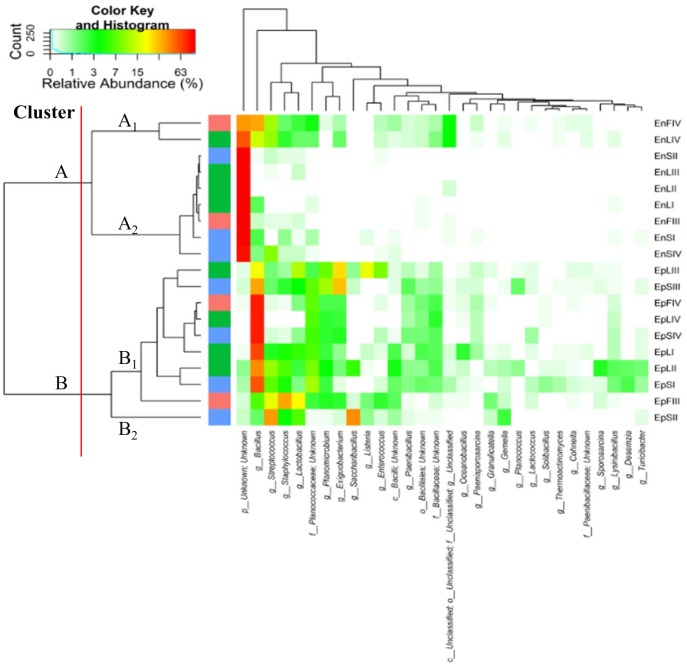
Pseudo heat map summarizing the relative abundances of the 30 most dominant genera in DNA samples directly extracted from epiphytic (Ep) and endophytic (En) *Firmicutes* found on leaves, stems and flowers of *Origanum vulgare* L. plant during the early vegetative (I), late vegetative (II), blooming (III) and full-flowering (IV) stages. The color key defines the percentages of OTUs in the samples.

**FIGURE 5:**
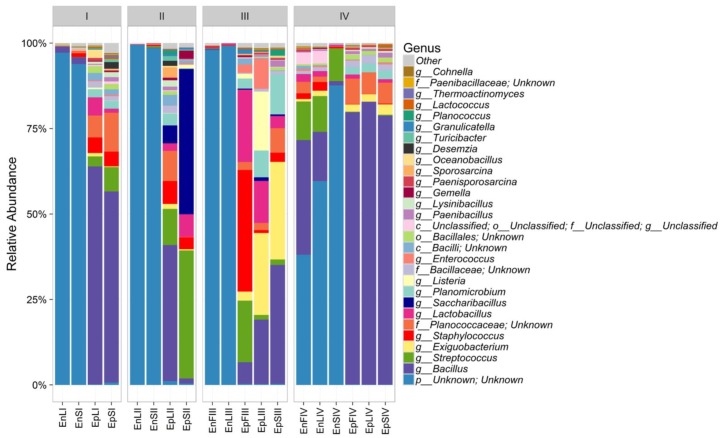
Relative abundance of epiphytic (Ep) and endophytic (En) *Firmicutes* genera found on leaves (L), stems (S) and flowers (F) of *Origanum vulgare* L. plant during the early vegetative (I), late vegetative (II), blooming (III) and full-flowering (IV) stages.

According to alpha- and beta-diversity, and considering the 30 most dominant genera of all the samples, endophytes and epiphytes distributed into two main clusters (A and B, respectively, **Figure 4**). Except for EnLIV and EnFIV that grouped in sub-cluster A1, all the endophytes gathered into the sub-cluster A2 regardless the plant organ and phenological stage. Similarly, all epiphytes grouped into sub-cluster B1, with the exception of EpSII. Occurring in most of the samples (90%), the core *Firmicutes* at genus level consisted of *Bacillus*, *Exiguobacterium*, *Streptococcus*, *Staphylococcus*, and *Lactobacillus*. OTUs belonging to *Enterococcus*, *Lactococcus*, *Lysinibacillus*, and *Planomicrobium* genera occurred in >70% of the samples (**Figure 5**). As epiphytes, *Bacillus* occurred in leaves (57.2 – 91.7%), stems (40.6 – 85.5%) and flowers (6.8 – 90.1%) and increased with the late phenological stages. At lower abundance, *Exiguobacterium* had a similar trend. Except for stage II, *Staphylococcus* and *Streptococcus* behaved oppositely. During time, OTUs of *Staphylococcus* and *Streptococcus* decreased in leaves (3.4 – 0.1% and 2.6 – 0%), S (2.3 – 0.1% and 5.3 – 0.3%) and flowers (35.8 – 0.03% and 19.9 – 0.24%). The genus *Lactobacillus* was present in leaves (up to 13.6%), S (up to 5.9%) and flowers (up to 24.2%). At stage IV, it disappeared from leaves. Although at low abundances, *Lysinibacillus* and *Planomicrobium* were stably present in leaves (up to 7.3 and 1.3%, respectively), stems (up to 10.3 and 0.9%) and flowers (up to 2.3 and 0.5%). Except for leaves at stage IV, *Lactococcus* (0.1 – 0.8% of relative abundance) was always found. *Enterococcus* reached the highest abundance in stems (0.4%) at stage II, and in L and flowers at stage III (8.2 and 1.9%, respectively) (**Figure 5**). As endophytes, *Bacillus*, *Exiguobacterium*, and *Staphylococcus* behaved similarly. They increased in leaves (up to 16.5, 1.5, and 2.6%, respectively) and flowers (up to 38.7, 0.7, and 2.3%), and decreased in stems (from 3.5 to 1.6, 0.1 to 0.01, and 1.2 to 0.6%) throughout the life cycle. During time, the presence of *Lactobacillus* and *Streptococcus* increased in leaves (1.5 and 9.9%, respectively), stems (0.7 and 9.7%) and flowers (2.4 and 10.8%). The highest abundance of *Lysinibacillus* in leaves (0.2%) and *Lactococcus*, *Enterococcus*, and *Planomicrobium* in flowers (0.2, 0.5, and 0.1%, respectively) was at stage IV. *Saccharibacillus*, *Listeria*, and *Planococcus* were detectable occasionally (**Figure 5**). When the assignment was possible, *Bacillus* sp. dominated (up to ca. 80%) the epiphyte species of all plant organs. *Staphylococcus epidermidis*, *Streptococcus* sp., *Exiguobacterium* sp. and *L. plantarum* followed. *Lc. lactis* was stable but at low abundance. *E. mundtii* was present occasionally. Leaves and flowers endophytes were dominated by *Bacillus* sp. (up to 35.5% in F), *Streptococcus thermophylus* (up to 10% in F), *St. epidermidis* (up to 2.7% in L), *L. plantarum* (up to 0.7% in F), *L. fuchuensis* (up to 0.7% in L) and *E. mundtii* (up to 0.5% in F). *B. endophyticus* was stable under both epiphyte (up to 3%) and endophyte (up to 2%) conditions. Epiphyte *Lactobacillus* group such as *Weissella confusa* and *Leuconostoc citreum* occurred occasionally.

### Chemistry of Essential Oils

Yields (%, v/w) in EOs from whole plant and plant organs were calculated based on dry matter weight. EOs of leaves and stems increased from stages I to II (2.9 and 0.1%, respectively). Flowers that appear at stage III had 5.8% of EOs. From stages II or III (in the case of F) onward, EOs yields of leaves, stems and flowers dropped from 2.5, 0.1 and 5.8% to 1.9, 0 and 4.6%, respectively. From stages I to IV, the EOs yield of the whole plant slightly increased (1.6 – 1.9%), which reflects the flowers appearance at stage III. The quantification and identification of EOs was after hydro-distillation of air-dried samples. In total, 51, 53, 49, and 42 components were identified in the four phenological stages. The most abundant were thymol and carvacrol. They accounted for ca. 74, 72, 83, and 85% of the total EOs identified during phenological stages. At lower abundances, precursor ρ-cymene and γ-terpinene were present (Supplementary Table S3). The content of thymol and carvacrol followed opposite trends. Thymol increased in the whole plant (16.16 ± 0.71% – 48.13 ± 0.05%), leaves (17.76 ± 0.12% – 24.94 ± 0.57%) and flowers (22.78 ± 0.56% – 33.22 ± 0.26%), but slightly decreased in stems (14.67 ± 0.77% – 13.65 ± 0.68%). Carvacrol decreased in the whole plant (45.59 ± 0.50% – 13.97 ± 0.01%), leaves (41.39 ± 0.23% – 31.37 ± 0.75%) and flowers (46.62 ± 1.28% – 37.51 ± 0.62%), with a concomitant increase (30.13 ± 0.27% – 34.35 ± 1.45%) in stems. The trend for ρ-cymene was similar to that found for carvacrol. Except for a slight decrease in the whole plant, the content of γ-terpinene increased during time. The further antimicrobial assays used chemically synthesized thymol and carvacrol because they were the most abundant EOs.

### Correlations Among Endophytic *Firmicutes* Genera and Essentials Oils

**FIGURE 6:**
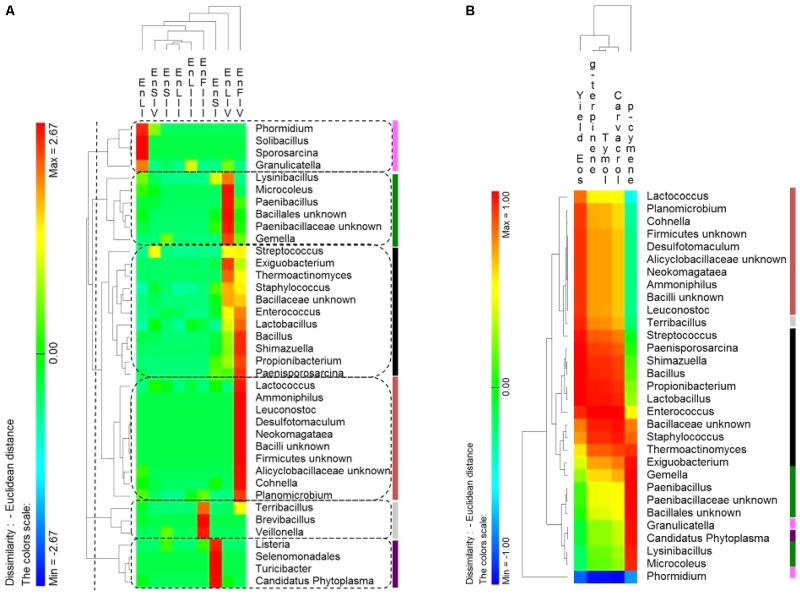
**(A)** Pseudo heat map summarizing the distribution of endophytic (En) *Firmicutes* genera on leaves, stems and flowers of *Origanum vulgare* L. plant during the early vegetative (I), late vegetative (II), blooming (III), and full-flowering (IV) stages. The color key defines the percentages of OTUs normalized row-by-row. Clusters are separated by the vertical dashed lines and highlighted by different colors. **(B)** Pseudo heatmap showing correlation (Pearson correlation) between the relative abundance of endophytic (En) *Firmicutes* genera found at the full-flowering (IV) stages, and the yield in Essential oils (Eos) and the most abundant EOs (%). A color scale ranging from a minimum of – 1 (negative correlation) to a maximum of 1 (positive correlation) was used. Clusters, as settled on the **(A)**, were highlighted by different colors.

The distribution of endophytic (En) *Firmicutes* genera showed a tissue- and a phenological stage-specific allocation (**Figure 6A**). Consequently, the correlations between the relative abundance of endophytic *Firmicutes* genera and the EOs content were investigated. Correlations were only found at the full-flowering stage (IV stage) between the relative abundance of genera and the EOs yields and percentage of thymol, carvacrol, ρ-cymene and γ-terpinene of stems, leaves, and flowers (**Figure 6B**). Clusters based on genera distribution almost matched with the clusters based on correlations with the EOs content (**Figure 6B**). The most positively correlated genera were *Bacillus*, *Enterococcus*, *Lactobacillus*, *Shimazuella*, *Propionibacterium*, and *Paenisporosarcina* (**Figure 6B**). These genera were mainly allocated into the leaves and flowers at IV stage (**Figure 6A**), matching with the highest yields of EOs.

### Antimicrobial Activity of Essential Oils and Lactic Acid Bacteria

The concentration of EOs during oregano life cycle was in the range 15.9 – 20.1 μg/g. The concentration of thymol and carvacrol for antimicrobial assays was in the range 3.9 – 1000 μg/ml. Therefore, total EOs extracted from oregano plant underwent to dilution or concentration to get the same interval of concentrations. The highest concentration (20.1 μg/g) of EOs found during oregano life cycle did not fully inhibit any bacterial strains. Decreases of cell yield (one to one a half log cycles) occurred for strains of *Leuc. citreum*, *E. faecium*, *E. mundtii*, *Lc. lactis*, *L. graminis*, and *L. rossiae*. The EOs concentration of 20.1 μg/g (data not shown) did not affect the cell yield of almost all strains of *L. plantarum*. Based on these results, the calculation of MIC referred to higher concentrations (**Table 2**). Only *L. plantarum* EpFIII_1_, EnFIII_9_ and EnSIV_6_ out of 23 strains tolerated thymol at a concentration higher than 1000 μg/ml. Eight, six, and four strains had values of MIC of 1000, 750, and 500 μg/ml, respectively. *Leuc. citreum* EnLI_9_ and *E. mundtii* EpSIV_7_ showed the highest sensitivity, being inhibited at 250 μg/ml. Only *L. plantarum* EpSI_4_, EpFIII_1,_ EnFIII_3_ and EpLI_13_ tolerated more than 1000 μg/ml of carvacrol. Except for *E. mundtii* EnSIV_3_ and EpSIV_7_, the values of MIC for the other strains were in the range of 500 – 1000 μg/ml. Regarding total EOs, the MIC for *L. plantarum* EnFIII_3_, EnFIII_9,_ EnSIII_5_, EnFIV_10_, and EnSIV_6_, *E. faecium* EpLI_20_, and *E. mundtii* EpSI_17_ was higher than 1000 μg/ml.

**Table 2:** Antimicrobial activities^a^ of essential oils extracted from the whole plant of *Origanum vulgare* L. at the early vegetative (I), late vegetative (II), blooming (III), and full flowering (IV) stages, thymol and carvacrol toward lactic acid bacterial strains.

Strain	Source of isolation (phenological stage)^b^	MIC (μg/ml)					
		I	II	III	IV	Thymol	Carvacrol
*Lactobacillus plantarum* EpSI_4_	Stems (I)	750	500	750	750	750	>1000
*Leuconostoc citreum* EnLI_9_	Leaves (I)	500	500	500	500	250	1000
*Enterococcus faecium* EpLI_16_	Leaves (I)	500	500	500	500	500	750
*L. plantarum* EnLIV_11_	Leaves (IV)	500	750	1000	750	1000	1000
*L. plantarum* EnFIII_3_	Flowers (III)	>1000	>1000	>1000	>1000	1000	>1000
*L. plantarum* EpFIII_1_	Flowers (III)	750	750	750	750	>1000	>1000
*L. plantarum* EnSIII_5_	Stems (III)	>1000	>1000	>1000	>1000	1000	1000
*Enterococcus mundtii* EnSIV_3_	Stems (IV)	500	750	750	500	500	250
*L. plantarum* EnFIII_9_	Flowers (III)	>1000	>1000	>1000	>1000	>1000	1000
*Lactococcus lactis* EpLIV_7_	Leaves (IV)	750	750	750	750	750	500
*E. mundtii* EpSIV_7_	Stems (IV)	>1000	250	750	750	250	250
*L. plantarum* EnFIV_10_	Flowers (IV)	>1000	>1000	>1000	>1000	1000	1000


^*a*^Minimum inhibitory concentration (MIC) values determined for the identified lactic acid bacterial strains; the data are from three independent experiments. ^*b*^Source of isolation and phenological stages are described in Section “Materials and Methods” and Supplementary Figure S4.

## Discussion

As humans and animals, plants do not interact randomly with microbes, but choose their specific interacting partners (Bulgarelli et al., 2012). Advances on plant–microbe interactions have drawn the attention to lactic acid bacteria as a new class of plant growth promoting microbes (Lamont et al., 2017). As a medicinal and aromatic plant, *Origanum vulgare* L. (oregano) containing high and variable levels of EOs, was chosen as suitable model to investigate the potential role of the endophyte-microbiome, especially lactic acid bacteria to drive the production of plant EOs. Consequently, the hypothesis of the colonization of plant tissues by endophytes resistant to these oils was verified (Checcucci et al., 2017).

A 16S rRNA gene-based high-throughput sequencing approach tracked the bacterial ecology during oregano life cycle, with particular focus on epiphyte and endophyte *Firmicutes*. A culture-dependent approach complemented the analyses. Lactic acid bacteria were only a part of the total cultivable bacteria. Total epiphyte bacteria were more abundant than the endophyte ones (Yadav et al., 2005). As shown for similar habitats, total aerobic bacterial endophytes decreased throughout oregano life cycle (James et al., 2002). This trend reflects the hostile changes of the inherent environmental conditions such as humidity and plant secondary metabolites (James et al., 2002).

Oregano organs harbored epiphyte and endophyte lactic acid bacteria, which increased throughout the life cycle, and corresponded to those usually found in most of the common plant matrices (Di Cagno et al., 2013). Flowers harbored the highest cell density of endophyte lactic acid bacteria, as confirmed both by culture-dependent and -independent approaches. The microbial colonization of flowers should reflect the bacterial dispersion by pollinators. Lactic acid bacteria are frequently associated with the gastrointestinal tracts of several insects. On the other hand, flowers offer nutrient-rich exudates as well as a protection from external stresses (Aleklett et al., 2014). Several species of lactic acid bacteria were identified throughout oregano life cycle, which corresponded to those most frequently identified from plant microbiotas (Di Cagno et al., 2013; Minervini et al., 2015). The culture-dependent approach allowed a clear differentiation between epiphytes and endophytes, and the reliable detection of the microbial succession. The highest diversity was at the early vegetative and full-flowering stages. Some species (e.g., *Leuconostoc citreum*, *Lactobacillus rossiae*, *Lactococcus lactis*, and *Lactobacillus graminis*) appeared occasionally, while others represented the core microbiota. *Lactobacillus plantarum* dominated in all plant organs over time, especially as endophyte. Strains of *L. plantarum* share phenotypic traits that determine their capacity to outcompete the contaminating bacterial biota (Minervini et al., 2010). Regardless the plant organ, *Enterococcus mundtii* flanked *L. plantarum*.

The high-throughput DNA sequencing approach was decisive for the epiphyte population only. It confirmed that the early vegetative and full-flowering stages harbored the largest microbial diversity. Proceeding with the plant growth, a number of species succeeded (Shade et al., 2013). Mainly the phyla *Proteobacteria*, *Actinobacteria*, and *Bacteroidetes* populated the oregano plant organs. Low abundances of *Firmicutes* and *Cyanobacteria* were present also in leaves and stems at different phenological stages. OTUs classified at genus level indicated *Methylobacterium*, *Sphingomonas*, *Rhizobium*, *Aurantimonas*, and *Pseudomonas* as the main epiphyte representatives. Although, epiphytes may reflect the environmental contamination, a stable presence of these genera throughout oregano life cycle confirmed the hypothetic protective role exerted by phyllosphere microbiota from diseases as well as promoting the plant growth by various mechanisms (Rastogi et al., 2013). These results were consistent with those reported for several agricultural crops (e.g., wheat, rice, apple, lettuce, and spinach) and naturally growing plants/trees (Bulgarelli et al., 2013). Epiphyte bacteria feature a high dynamism in these unusual open systems (Hirano and Upper, 2000), and only a few genera persist in these habitats (Lindow and Brandl, 2003). The pigmentation of *Methylobacterium*, *Sphingomonas*, and *Pseudomonas* is important for protection against UV radiation (Sundin, 2002; Lindow and Brandl, 2003), which is one of the most noticeable stresses for the epiphyte population. *Methylobacterium* and *Sphingomonas* accounted for ca. 50% of the oregano leave and stem microbiotas (Knief et al., 2010). The flower population consisted mainly of *Serratia* and *Acinetobacter*. These genera were already isolated as flower inhabitants, being responsible for pistil spoilage (Aleklett et al., 2014).

Targeting *Firmicutes*, the epiphyte diversity was also the largest at early vegetative and full-flowering stages. Zooming on the epiphyte lactic acid bacteria, the largest diversity coincided with flowers appearance (blooming stage). *Bacillus*, *Exiguobacterium*, *Staphylococcus*, *Lactobacillus*, and *Streptococcus* were the core genera. Previously, all these genera were identified as epiphytes and endophytes of forage crops (Pang et al., 2012; Gaggìa et al., 2013) or wheat plants (Minervini et al., 2015). The relative abundance at genus level depended on plant organ and, especially, phenological stage. Microbes randomly emerge from neighboring environments, but the plant regulates their survival (Berlec, 2012). *Bacillus* was the most abundant epiphyte and endophyte genus in all the oregano organ plants probably because of its capacity to tolerate EOs. *Bacillus* was isolated as the dominant endophyte in pepper-rosemary, which is the medicinal plant with the highest level of EOs (da Silva et al., 2013). *Bacillus*, *Exiguobacterium*, and *Staphylococcus* were isolated also from halotolerant plant species, where they act as nitrogen fixers, phosphate solubilizes and/or auxin producers (Bian et al., 2011). The relative abundance of epiphyte and endophyte *Bacillus* and *Exiguobacterium* increased throughout the plant life cycle. The trend for *Staphylococcus* and *Streptococcus* was the opposite. *Lactobacillus* was stably isolated as epiphyte and endophyte. In agreement with the culture-dependent approach, its abundance increased during time. Overall, epiphyte *Lactobacillus* seemed to prefer leaves and, especially, flowers. As endophytes, they distributed almost equally leaves, stems and flowers. *Lysinibacillus*, *Paenibacillus*, *Planomicrobium*, *Pediococcus*, *Leuconostoc*, and *Weissella* were present occasionally at low relative abundances. First, lactic acid bacteria were isolated as components of the epiphyte and endophyte populations in a medicinal and aromatic plant. *E. mundtii*, *L. plantarum*, *Pediococcus pentosaceus*, *Lc. lactis* and *Streptococcus* sp. were the most abundant species. In agreement with the culture-dependent approach, *L. plantarum* was the only endophyte lactic acid bacterium identified throughout all the phenological stages. The same high-throughput DNA sequencing approach showed that *L. plantarum* stably persisted within the endophyte population of the wheat grain throughout the life cycle (Minervini et al., 2015). Despite the obvious isolation of *L. plantarum* from plant matrices, it is one of the best-characterized plant-associated bacteria, which transform a multitude of plant-derived raw materials through fermentation (De Vuyst et al., 2009). Its remarkable adaptability, wide industrial utility and potent impact on animal and plant physiology have made *L. plantarum* a model organism of significant interest to the scientific community.

With variations among the organs, oregano plant had the highest yield of EOs at the full-flowering stage due to the flowers presence. Previous reports (Rohloff et al., 2005; Kizil et al., 2010) confirmed that peppermint, *Satureja rechingeri*, species of the *Lamiaceae* family, and oregano had the highest yields of EOs at the full-flowering stage. Thymol and carvacrol, flanked by their precursors ρ-cymene and γ-terpinene, were the most abundant EOs. At the full-flowering stage, *Firmicutes* genera distribution seemed reflect the different EOs contents of stems, leaves and flowers. Several authors highlighted specific interactions between plant microbiota and plant EOs and other volatiles (Banchio et al., 2008; Checcucci et al., 2017; Burdon et al., 2018). In the context of flowers, volatiles could affect the microbiota composition, controlling bacteria having negative effects on pollinator-interactions (Burdon et al., 2018). Anyway, not all plant–bacteria interactions are antagonistic, and some bacteria are able to metabolize volatiles (Del Giudice et al., 2008). We found *Bacillus*, *Enterococcus*, *Lactobacillus*, *Shimazuella*, *Propionibacterium*, and *Paenisporosarcina* as the most positively correlated with the EOs content, which were mainly allocated into the flowers. Plant–bacteria interactions mediated by EOs and other volatiles have been already investigated for several species belonging to *Bacillus* and *Pseudomonas* genera (Banchio et al., 2008; Checcucci et al., 2017; Burdon et al., 2018), but no previous studies considered *Enterococcus* and *Lactobacillus*, although lactic acid bacteria are present in the phyllosphere, endosphere and rhizosphere of many plants. Under our experimental conditions, the EOs content of oregano plant seemed to do not markedly interfere with the lactic acid bacteria survival. As showed by the positive correlation analysis, the distribution of the endophyte lactic acid bacteria within oregano agreed with the EOs concentration. Values of MIC higher than 1000 μg/ml supported our hypothesis that the persistence of several *L. plantarum* strains as endophytes reflected their ability to tolerate these two main oregano EOs. Recently, the adaptation and the colonization of endophytes in *Thymus* spp. was correlated with the pattern of resistances to the EOs present in leaf glandular tissue, confirming the EOs as one of the selective forces shaping endophytic community composition (Checcucci et al., 2017).

This study gives a picture of the epiphyte and endophyte populations of oregano organs throughout the life cycle. It demonstrates the stable persistence of a lactic acid bacteria core, which was dominated by *L. plantarum* strains. The colonization by lactic acid bacteria was the highest in flowers. Their presence followed the maturity of plant, signing the stage of the full flowering. EOs of oregano plant were profiled, showing that the yield of EOs for leaves and flowers is a driver affecting the level of most of the endophytes. The positive correlation among entophyte lactic acid bacteria and the yield of EOs and the concentration of the most abundant EOs seems confirm the hypothesis that the colonization within plant niches such as flowers may be regulated by mechanisms driving the synthesis of the secondary metabolites. Due to their antimicrobial activity, endophyte lactic acid bacteria may be also considered the storehouse of secondary metabolites that can enhance the resistance of oregano to pests. This study offers a preliminary but promising example of the biotechnological potential of lactic acid bacteria isolated from oregano plant, which opens to further investigations on oregano bioprocessing of bioactive phytochemicals and that may have repercussions also on other medicinal and aromatic plants.

## Author Contributions

EP carried out the experiments. RC discussed the results and wrote the manuscript. WT carried out the experiments on the EOs analysis and related data elaboration. PF carried out the experiments on the MIC and related data elaboration. GM directed the experimental phase on plants and agronomic features. MG ideated the study and made funds available for the research costs.

## Conflict of Interest Statement

The authors declare that the research was conducted in the absence of any commercial or financial relationships that could be construed as a potential conflict of interest.

## Footnotes

1 https://www.ezbiocloud.net/
