# Supplementary material for: Dynamic and Assembly of Epiphyte and Endophyte Lactic Acid Bacteria During the Life Cycle of Origanum vulgare L
Source: Front Microbiol. 2018 Jun 26;9:1372. doi: 10.3389/fmicb.2018.01372 (PMC6029521; doi:10.3389/fmicb.2018.01372)
Supplement: Supplementary file 1 [file Table_1.pdf]

Supplementary Material

Dynamic and Assembly of Epiphyte and Endophyte Lactic Acid Bacteria during the Life Cycle of *Origanum vulgare* L.

Erica Pontonio[1], Raffaella Di Cagno[2*], Waed Tarraf[3], Pasquale Filannino[1], Giuseppe De Mastro[3], Marco Gobbetti[2]

*** Correspondence:** Corresponding Author: Raffaella.dicagno@unibz.it

Table S1. Number of sequences analyzed, observed diversity, and abundance-based coverage estimator for 16S rRNA amplification from epiphytic (Ep) fraction of leaves (L), stems (S) and flowers (F) of *Origanum vulgare* L. plant during the early vegetative (I), late vegetative (II), blooming (III) and full-flowering (IV) stages.

	Richness			Diversity index	
**Sample**	**No. OTU**	**Chao 1 richness**	**ACE**	**Shannon**	**Simpson**
EpLI	162	165.7	165.7	3.2	0.91
EpLII	150	152.1	153.6	3.0	0.89
EpLIII	139	140.6	141.9	2.5	0.76
EpLIV	192	195.9	198.6	3.5	0.92
EpSI	130	142.4	146.6	3.1	0.91
EpSII	89	100.1	97.1	2.9	0.91
EpSIII	144	151.0	150.4	3.2	0.91
EpSIV	145	170.2	164.1	3.0	0.91
EpFIII	63	63.1	63.6	0.48	0.14
EpFIV	80	96.5	101.0	1.6	0.59

Abbreviations: OTU, operational taxonomic unit; ACE, abundance-based coverage estimator. Chao1 richness, Shannon diversity, and ACE were calculated with Qiime at the 4% distance level. Further details were included in Material and methods.

Table S2. Number of sequences analyzed, observed diversity, and abundance-based coverage estimator for a fragment of the 16S rRNA inside the phylum of *Firmicutes* from epiphytic (Ep) and endophytic (En) fractions of leaves (L), stems (S) and flowers (F) of the *Origanum vulgare* L. plant during the early vegetative (I), late vegetative (II), blooming (III) and full-flowering (IV) stages.

	Richness			Diversity index	
**Sample**	**No. OTU**	**Chao 1 richness**	**ACE**	**Shannon**	**Simpson**
**Epiphytic fraction**					
EpLI	97	108.1	104.4	3.1	0.9
EpLII	81	82.5	83.9	3.3	0.9
EpLIII	93	93.8	95.0	2.9	0.9
EpLIV	81	90.7	90.7	2.3	0.8
EpSI	98	99.0	98.8	3.0	0.9
EpSII	65	72.0	70.2	1.8	0.7
EpSIII	75	85.0	77.5	2.8	0.9
EpSIV	95	100.2	104.7	2.5	0.8
EpFIII	60	69.3	67.0	2.4	0.8
EpFIV	106	110.2	112.3	2.5	0.8
**Endophytic fraction**					
EnLI	57	59.0	60.0	0.26	0.06
EnLII	18	18.0	18.0	0.04	0.01
EnLIII	23	25.1	28.7	0.07	0.02
EnLIV	57	57.0	57.0	1.78	0.63
EnSI	46	46.8	48.4	0.41	0.12
EnSII	35	38.0	40.9	0.13	0,03

Table S2 (Continued)

	Richness			Diversity index	
**Sample**	**No. OTU**	**Chao 1 richness**	**ACE**	**Shannon**	**Simpson**
**Endophytic fraction**					
EnSIII	-	-	-	-	-
EnSIV	43	43.5	43.9	0.54	0.22
EnFIII	27	37.0	37.5	0.16	0,04
EnFIV	80	81.5	83.1	2.45	0.82

Abbreviations: OTU, operational taxonomic unit; ACE, abundance-based coverage estimator. Chao1 richness, Shannon diversity, and ACE were calculated with Qiime at the 4% distance level. Further details were included in Material and methods.

Table S3. Essential oil composition (%) extracted from *Origanum vulgare* L. whole plant (WP), leaves (L), stems (S), and flowers (F) during the early vegetative (I), late vegetative (II), blooming (III) and full-flowering (IV) stages.

				I			II			III			IV		
**Peak**	**Compounds**	**R.T.**	**RI[a]**	**Whole plant**	**Leave**	**Stem**	**Whole plant**	**Leave**	**Stem**	**Whole plant**	**Leave**	**Flower**	**Whole plant**	**Leave**	**Flower**
**Monoterpenes hydrocarbons**				19.84±3.58^{c}	25.23±0.26^{abc}	22.95±1.57^{bc}	20.03±1.80^{c}	22.16±0.19^{bc}	15.97±1.11^{d}	22.55±0.31^{bc}	27.23±0.33^{ab}	21.13±1.69^{c}	27.65±0.44^{a}	28.17±1.95^{a}	22.28±0.68^{bc}
**1**	α-thujene	7.35	929	0.16±0.00	0.07±0.02	0.26±0.03	0.15±0.01	-	-	-	0.01±0.00	0.12±0.02	-	-	0.03±0.01
**2**	α-pinene	7.58	937	0.32±0.06	0.42±0.01	0.26±0.04	0.51±0.05	0.50±0.03	0.24±0.03	0.46±0.03	0.54±0.01	0.43±0.06	0.46±0.01	0.41±0.06	0.48±0.03
**3**	Camphene	8.09	952	0.15±0.03	0.14±0.00	0.10±0.02	0.38±0.01	0.32±0.00	0.29±0.02	0.26±0.01	0.38±0.01	0.15±0.02	0.09±0.01	0.17±0.02	0.13±0.00
**4**	β-pinene	9.08	979	-	0.04±0.01	0.05±0.02	0.06±0.00	0.02±0.01	-	0.02±0.00	0.03±0.02	0.04±0.03	-	-	-
**5**	β-myrcene	9.62	992	0.78±0.14	1.04±0.01	0.59±0.19	1.03±0.01	1.06±0.00	0.41±0.14	0.94±0.02	0.95±0.03	1.09±0.14	1.03±0.01	0.68±0.07	1.11±0.05
**6**	α-phellandrene	10.12	1005	0.17±0.03	0.21±0.00	0.11±0.04	0.21±0.03	0.21±0.03	0.12±0.01	0.20±0.02	0.17±0.01	0.22±0.05	0.21±0.00	0.13±0.01	0.21±0.02
**7**	δ^3-carene	10.36	1011	0.03±0.02	0.05±0.01	0.04±0.02	0.05±0.00	0.05±0.00	-	0.05±0.01	0.06±0.00	0.05±0.01	-	0.04±0.00	0.05±0.00
**8**	α-terpinene	10.61	1018	1.44±0.26	1.77±0.13	1.58±0.06	1.41±0.33	1.50±0.29	1.06±0.00	1.35±0.13	1.28±0.15	1.85±0.53	1.91±0.19	0.71±0.06	1.95±0.07
**9**	ρ-cymene	10.93	1027	3.55±0.64	4.98±1.01	3.62±0.37	3.62±0.16	4.53±1.20	2.39±0.45	7.26±0.88	12.03±0.79	4.13±1.32	9.28±0.19	16.32±0.30	4.74±0.15
**10**	β-phellandrene	11.10	1031	0.24±0.04	0.31±0.02	0.21±0.01	0.34±0.00	0.33±0.01	0.17±0.03	0.34±0.01	0.36±0.01	0.32±0.02	0.38±0.03	0.30±0.01	0.35±0.00
**11**	Cis-ocimene ((Z)-β-ocimene)	11.49	1041	0.51±0.09	0.80±0.01	0.58±0.01	1.28±0.05	1.31±0.04	0.64±0.04	0.69±0.01	0.64±0.02	0.03±0.00	-	0.15±0.01	-

Table S3 (Continued)

Peak	Compounds	R.T.	RI[a]	I			II			III			IV		
				**Whole plant**	**Leave**	**Stem**	**Whole plant**	**Leave**	**Stem**	**Whole plant**	**Leave**	**Flower**	**Whole plant**	**Leave**	**Flower**
**Monoterpenes hydrocarbons**				19.84±3.58[c]	25.23±0.26[abc]	22.95±1.57[bc]	20.03±1.80[c]	22.16±0.19[bc]	15.97±1.11[d]	22.55±0.31[bc]	27.23±0.33[ab]	21.13±1.69[c]	27.65±0.44[a]	28.17±1.95[a]	22.28±0.68[bc]
**12**	Trans-ocimene ((E)-β-ocimene)	11.92	1051	0.08±0.01	0.11±0.00	0.10±0.03	0.14±0.02	0.14±0.01	0.07±0.01	0.11±0.00	0.14±0.02	0.06±0.01	0.14±0.01	0.21±0.06	0.08±0.00
**13**	γ-terpinene	12.36	1062	12.34±2.24	15.20±0.68	15.46±1.22	10.73±1.27	12.06±1.06	10.49±0.82	10.77±0.53	10.52±0.83	12.52±2.12	14.04±0.65	8.89±1.38	13.05±0.64
**14**	α-terpinolene	13.63	1089	0.05±0.01	0.08±0.02	-	0.12±0.01	0.12±0.00	0.10±0.02	0.11±0.02	0.13±0.00	0.09±0.00	0.10±0.02	0.14±0.01	0.10±0.01
**Oxygenated monoterpene**				5.20±0.03[f]	5.45±0.11[f]	9.00±0.38[c]	8.32±0.05 [cd]	7.44±0.02d[e]	12.85±1.71[a]	5.60±0.18[f]	6.76±0.16e[f]	2.85±0.13[g]	5.24±0.06[f]	10.88±0.10[b]	3.46±0.09[g]
**17**	1-octen-3-ol	9.16	981	0.71±0.05	0.62±0.01	0.41±0.03	0.68±0.02	0.71±0.02	0.49±0.24	0.77±0.01	1.19±0.05	0.18±0.00	0.83±0.01	1.37±0.01	0.23±0.02
**18**	3-octanone	9.44	988	0.41±0.01	0.41±0.01	0.59±0.07	0.42±0.00	0.39±0.01	0.45±0.03	0.29±0.00	0.41±0.00	0.07±0.01	0.27±0.00	0.40±0.00	0.07±0.01
**19**	3-octanol	9.79	996	-	-	0.01±0.00	0.03±0.01	0.03±0.01	0.07±0.01	-	0.04±0.01	-	-	0.04±0.01	-
**20**	1,8-cineole	11.22	1034	-	-	-	0.01±0.00	-	-	-	0.01±0.00	-	-	-	0.01±0.00
**21**	Linalool	14.14	1100	0.07±0.02	0.05±0.00	0.15±0.02	0.17±0.01	0.21±0.00	0.62±0.12	0.14±0.01	0.20±0.02	0.05±0.00	0.13±0.05	0.30±0.05	0.08±0.01
**22**	Borneol	17.05	1168	0.31±0.06	0.19±0.05	0.42±0.03	0.91±0.02	0.67±0.03	2.67±0.51	0.61±0.06	0.78±0.07	0.29±0.06	-	0.18±0.03	0.13±0.09
**23**	Terpinen-4-ol	17.56	1179	0.21±0.02	0.21±0.04	0.31±0.10	0.30±0.06	0.28±0.05	0.35±0.10	0.31±0.07	0.29±0.02	0.32±0.05	0.36±0.08	0.37±0.11	0.33±0.04
**24**	α-terpineol	18.18	1191	-	0.05±0.01	0.04±0.00	0.06±0.01	0.05±0.00	0.05±0.02	0.06±0.00	0.07±0.00	0.06±0.00	0.09±0.02	0.09±0.01	0.08±0.01

Table S3 (Continued)

				I			II			III			IV		
**Peak**	**Compounds**	**R.T.**	**RI^{a}**	**Whole plant**	**Leave**	**Stem**	**Whole plant**	**Leave**	**Stem**	**Whole plant**	**Leave**	**Flower**	**Whole plant**	**Leave**	**Flower**
**Oxygenated monoterpene**				5.20±0.03^{f}	5.45±0.11^{f}	9.00±0.38^{c}	8.32±0.05 ^{cd}	7.44±0.02d^{e}	12.85±1.71^{a}	5.60±0.18^{f}	6.76±0.16e^{f}	2.85±0.13^{g}	5.24±0.06^{f}	10.88±0.10^{b}	3.46±0.09^{g}
**25**	Cis-dihydrocarvone	18.43	1197	-	-	0.03±0.00	0.02±0.00	0.03±0.00	0.08±0.01	-	-	0.03±0.01	-	-	-
**26**	Bornyl formate	19.37	1219	0.20±0.04	0.22±0.01	0.09±0.02	0.24±0.00	0.28±004	0.15±0.02	0.25±0.04	0.30±0.05	0.28±0.06	0.26±0.05	0.23±0.07	0.29±0.00
**15**	Thymol methyl ether	20.15	1237	0.25±0.00	0.18±0.01	0.44±0.01	1.29±0.00	1.00±0.00	1.71±0.29	0.75±0.03	1.13±0.01	0.11±0.00	0.82±0.00	1.82±0.00	0.31±0.01
**16**	Carvacrol-methyl-ether	20.55	1247	3.02±0.02	3.46±0.01	6.26±0.14	4.05±0.00	3.78±0.05	5.70±1.69	2.25±0.00	2.23±0.01	1.07±0.02	2.48±0.01	6.01±0.00	1.81±0.03
**27**	Bornyl acetate	22.41	1287	-	-	-	0.14±0.03	-	0.32±0.01	0.07±0.03	0.10±0.02	0.02±0.01	-	0.05±0.00	0.06±0.01
**28**	Thymyl acetate	25.38	1357	-	-	-	-	-	-	-	-	0.10±0.01	-	-	-
**29**	Carvacryl acetate	26.18	1375	0.02±0.00	-	0.23±0.04	-	-	0.19±0.03	0.09±0.00	-	0.26±0.04	-	-	0.07±0.01
**Phenolic monoterpenes**				61.75±1.21^{c}	59.15±0.35^{d}	44.80±1.04^{g}	62.71±0.80^{c}	62.48±0.41^{c}	48.00±0.77^{f}	65.13±0.41^{b}	59.47±0.62^{d}	69.40±1.84^{a}	62.10±0.06^{c}	56.31±1.32^{e}	70.73±0.88^{a}
**30**	Thymol	22.78	1295	16.16±0.71	17.76±0.12	14.67±0.77	32.60±0.21	23.67±0.06	13.65±0.68	34.79±0.37	34.67±0.28	22.78±0.56	48.13±0.05	24.94±0.57	33.22±0.26
**31**	Carvacrol	23.19	1304	45.59±0.50	41.39±0.23	30.13±0.27	30.11±0.59	38.81±0.35	34.35±1.45	30.04±0.03	24.79±0.34	46.62±1.28	13.97±0.01	31.37±0.75	37.51±0.62
**Sesquiterpene hydrocarbons**				9.40±0.24^{c}	9.26±0.57^{c}	23.41±0.44^{a}	7.48±0.80^{d}	7.62±0.67^{d}	19.98±1.17^{b}	6.80±0.17^{d}	6.44±0.16^{d}	6.61±0.32^{d}	4.84±0.32^{e}	4.47±0.41^{e}	3.55±0.09^{e}
**32**	α-ylangene	26.09	1373	0.07±0.01	0.09±0.04	0.08±0.03	-	-	0.05±0.01	-	-	-	-	0.06±0.01	-
**33**	α-copaene	26.28	1377	0.14±0.05	0.09±0.03	0.20±0.03	-	-	0.16±0.05	0.06±0.01	-	-	-	0.09±0.02	-

Table S3 (Continued)

				I			II			III			IV		
**Peak**	**Compounds**	**R. T.**	**RI[a]**	**Whole plant**	**Leave**	**Stem**	**Whole plant**	**Leave**	**Stem**	**Whole plant**	**Leave**	**Flower**	**Whole plant**	**Leave**	**Flower**
**Sesquiterpene hydrocarbons**				9.40±0.24^{c}	9.26±0.57^{c}	23.41±0.44^{a}	7.48±0.80^{d}	7.62±0.67^{d}	19.98±1.17^{b}	6.80±0.17^{d}	6.44±0.16^{d}	6.61±0.32^{d}	4.84±0.32^{e}	4.47±0.41^{e}	3.55±0.09^{e}
**34**	β-bourbonene	26.67	1386	0.08±0.01	0.12±0.01	0.07±0.04	0.04±0.00	-	0.04±0.00	0.08±0.00	0.08±0.01	-	-	0.16±0.04	-
**35**	Trans-caryophyllene	28.10	1420	0.67±0.12	0.64±0.09	1.72±0.07	1.85±0.52	1.69±0.34	4.14±0.45	1.18±0.14	0.95±0.16	1.40±0.30	1.13±0.21	0.41±0.10	0.44±0.05
**36**	Aromadendrene	28.90	1440	0.05±0.01	0.07±0.01	0.14±0.03	0.06±0.04	0.06±0.05	0.26±0.00	0.08±0.00	0.07±0.01	0.06±0.03	-	-	-
**37**	α-humulene	29.50	1455	0.05±0.01	0.06±0.00	0.11±0.02	0.23±0.05	0.18±0.03	0.45±0.06	0.13±0.00	0.11±0.01	0.18±0.03	0.20±0.02	0.06±0.02	0.04±0.01
**38**	allo-aromadendrene	29.83	1463	-	0.03±0.02	0.16±0.04	0.03±0.01	0.03±0.01	0.10±0.01	-	0.02±0.00	-	-	-	-
**39**	Trans-cadina-1(6),4-diene	30.35	1475	-	0.05±0.02	0.39±0.03	0.04±0.00	-	-	-	-	-	-	-	-
**40**	γ-muurolene	30.47	1478	0.47±0.02	0.66±0.17	1.84±0.22	0.20±0.01	0.21±0.05	0.69±0.13	0.17±0.05	0.11±0.04	0.09±0.01	0.25±0.10	0.11±0.04	0.14±0.04
**41**	β-cadinene	30.61	1481	0.04±0.01	0.04±0.01	0.16±0.00	-	-	0.07±0.02	-	-	-	-	-	-
**42**	α-elemene	31.21	1495	0.41±0.07	0.43±0.11	0.99±0.16	0.26±0.01	0.27±0.05	0.90±0.17	0.20±0.05	0.16±0.03	0.14±0.05	0.15±0.01	0.10±0.02	0.10±0.01
**43**	α-muurolene	31.43	1500	0.17±0.08	0.10±0.03	0.57±0.13	0.05±0.02	-	0.22±0.04	0.06±0.02	-	-	-	-	-
**44**	β-bisabolene	31.77	1510	4.28±0.22	3.84±0.03	9.65±0.55	3.45±0.00	3.70±0.00	8.18±1.36	3.73±0.00	4.24±0.03	4.05±0.16	1.78±0.07	2.68±0.16	2.04±0.00
**45**	γ-cadinene	31.98	1515	0.88±0.16	0.91±0.21	2.01±0.19	0.40±0.03	0.44±0.09	1.29±0.03	0.36±0.01	0.25±0.00	0.21±0.02	0.43±0.04	0.29±0.07	0.25±0.03

Table S3 (Continued)

				I			II			III			IV		
**Peak**	**Compounds**	**R. T.**	**RI**[a]	**Whole plant**	**Leave**	**Stem**	**Whole plant**	**Leave**	**Stem**	**Whole plant**	**Leave**	**Flower**	**Whole plant**	**Leave**	**Flower**
**Sesquiterpene hydrocarbons**				9.40±0.24^{c}	9.26±0.57^{c}	23.41±0.44^{a}	7.48±0.80^{d}	7.62±0.67^{d}	19.98±1.17^{b}	6.80±0.17^{d}	6.44±0.16^{d}	6.61±0.32^{d}	4.84±0.32^{e}	4.47±0.41^{e}	3.55±0.09^{e}
**46**	δ-cadinene	32.36	1525	1.70±0.31	1.88±0.21	4.57±0.12	0.76±0.15	0.89±0.15	3.03±0.17	0.69±0.01	0.45±0.02	0.42±0.06	0.81±0.00	0.42±0.03	0.50±0.02
**47**	Cadina-1,4-diene	32.70	1534	0.04±0.00	-	0.10±0.06	-	-	0.05±0.02	-	-	-	-	-	-
**48**	α-cadinene	32.90	1540	0.26±0.05	0.25±0.08	0.45±0.18	0.08±0.00	0.10±0.02	0.23±0.05	0.06±0.02	-	0.03±0.02	0.08±0.03	0.02±0.01	0.04±0.02
**49**	Cis-alpha-bisabolene	33.12	1545	-	-	0.14±0.04	0.03±0.01	0.03±0.01	0.12±0.02	-	-	0.04±0.01	0.02±0.00	0.03±0.00	0.01±0.00
**Oxygenated sesquiterpene**				0.34±0.08^{c}	0.49±0.05^{c}	1.61±0.13^{b}	0.23±0.01^{d}	-	1.53±0.16^{b}	0.16±0.01^{d}	0.10±0.01^{d}	-	0.07±0.02^{d}	0.15±0.05^{d}	-
**50**	Spathulenol	34.45	1579	-	-	0.05±0.01	-	-	-	-	0.10±0.01	-	-	0.15±0.05	-
**51**	α-muurolol	36.89	1644	-	0.08±0.01	0.28±0.01	0.07±0.01	-	0.61±0.08	-	-	-	-	-	-
**52**	α-eudesmol	37.36	1657	0.08±0.01	0.15±0.03	0.53±0.04	0.05±0.00	-	0.37±0.02	0.07±0.01	-	-	-	-	-
**53**	α-bisabolol	38.40	1684	0.26±0.05	0.26±0.08	0.75±0.08	0.11±0.00	-	0.55±0.10	0.09±0.03	-	-	0.07±0.02	-	-
**Total (%)**				96.82	99.64	100.00	98.77	99.71	98.32	99.94	99.99	99.99	99.89	100.00	100.00

[a]RI**:** Retention index on a HP-5 MS column; results are the mean of three replicates ± SD ($P<0.05$). Values within a column with different superscript letters are significantly different ($P<0.05$).
