# Supplementary material for: Dynamic and Assembly of Epiphyte and Endophyte Lactic Acid Bacteria During the Life Cycle of Origanum vulgare L
Source: Front Microbiol. 2018 Jun 26;9:1372. doi: 10.3389/fmicb.2018.01372 (PMC6029521; doi:10.3389/fmicb.2018.01372)
Supplement: Supplementary file 2 [file Image_1.pdf]

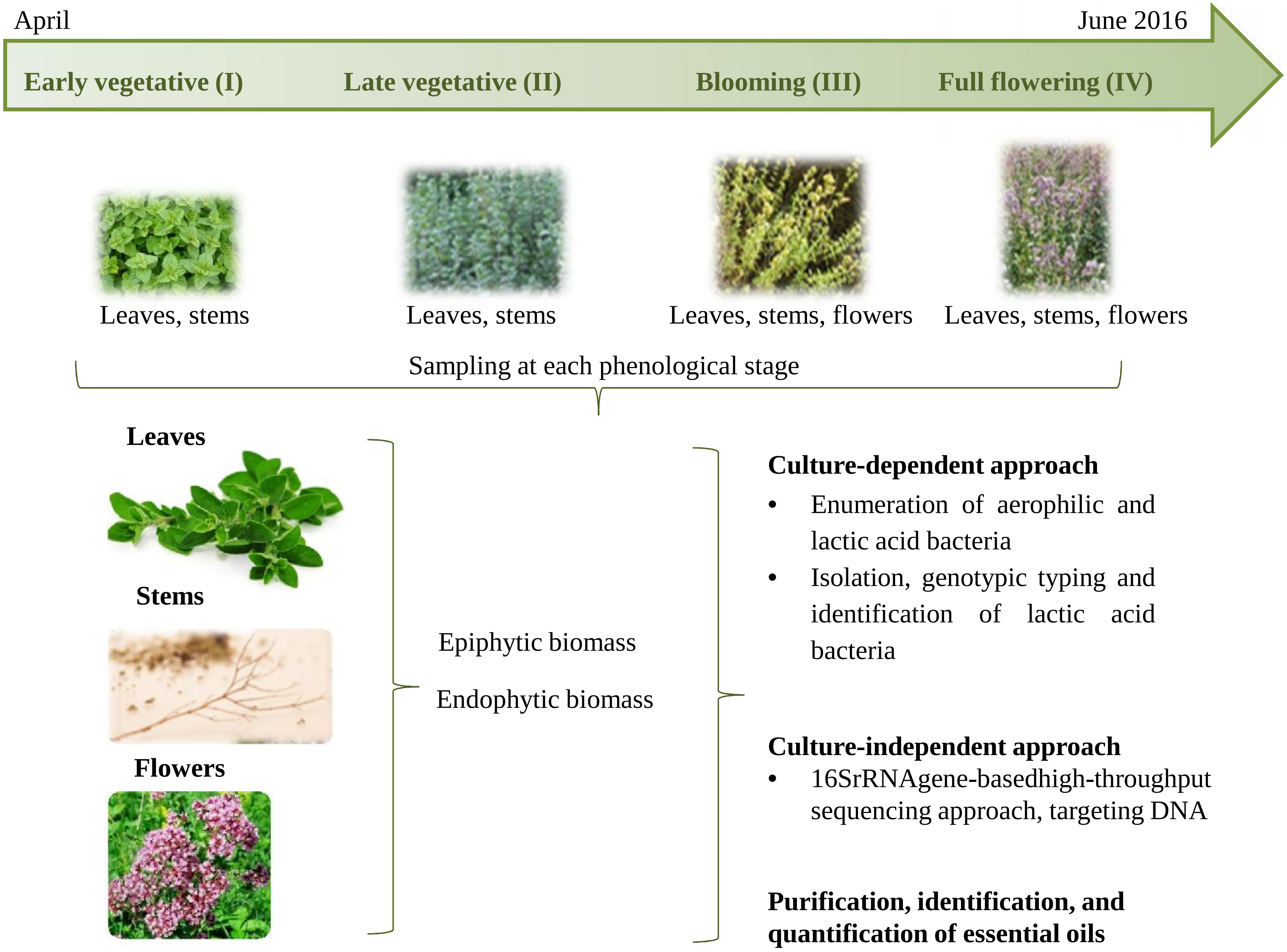
April
June 2016
Early vegetative (I)
Late vegetative (II)
Blooming (III)
Full flowering (IV)
Leaves, stems
Leaves, stems
Leaves, stems, flowers
Leaves, stems, flowers
Sampling at each phenological stage
Leaves
Stems
Flowers
Epiphytic biomass
Endophytic biomass
Culture-dependent approach
• Enumeration of aerophilic and lactic acid bacteria
• Isolation, genotypic typing and identification of lactic acid bacteria
Culture-independent approach
• 16SrRNAgene-basedhigh-throughput sequencing approach, targeting DNA
Purification, identification, and quantification of essential oils
